# Association between fetal exposure to the Chinese famine and cognitive decline in adulthood: a retrospective cohort study

**DOI:** 10.3389/fnut.2025.1532721

**Published:** 2025-06-13

**Authors:** Shuai Xiang, Yixuan Li, Bingzi Dong, Haochen Chi, Yangang Wang, Shanglong Liu

**Affiliations:** ^1^Department of Gastrointestinal Surgery, The Affiliated Hospital of Qingdao University, Qingdao, China; ^2^Department of Pancreatic and Gastric Surgery, National Cancer Center, National Clinical Research Center for Cancer, Cancer Hospital, Chinese Academy of Medical Sciences and Peking Union Medical College, Beijing, China; ^3^Department of Endocrinology, The Affiliated Hospital of Qingdao University, Qingdao, China; ^4^Department of Neurology, Qingdao Municipal Hospital, Qingdao University, Qingdao, China

**Keywords:** Chinese famine, malnutrition, cognitive function, fetal exposure, CHARLS

## Abstract

**Background:**

The Great Chinese Famine in the 1960s represents a significant historical event with potential long-term health consequences. This study aims to investigate the impact of famine exposure during different developmental stages (fetal, preschool, school-age, and unexposed) on cognitive function in adulthood.

**Methods:**

A retrospective cohort study was conducted among 4,067 participants from the China Health and Retirement Longitudinal Study (CHARLS) database. Participants’ famine exposure histories were categorized based on birthdates and famine severity, and their cognitive function was assessed in adulthood. Multiple linear regression analysis was used to explore the relationships between famine exposure during different life stages and average cognitive score from 2011 to 2015.

**Results:**

Our study showed that fetal exposure to famine was significantly associated with lower cognitive function scores in adulthood, compared to individuals who were not exposed or exposed during other life stages (preschool or school-age). After adjusting for confounding factors, the fetal exposed group showed a statistically significant decrease in global cognition (*β* = −0.60, 95% CI: −0.95, −0.25), episodic memory (*β* = −0.25, [95% CI: −0.42, −0.07]), and executive function (*β* = −0.36, [95% CI: −0.61, −0.10]) compared to the non-exposed group. Furthermore, participants from severely affected famine areas exhibited significantly lower cognitive function scores compared to those from less severely affected famine areas after adjusting for all confounding factors.

**Conclusion:**

Fetal exposure to severe famine was associated with reduced cognitive performance in adulthood. This study provided new evidence for developing prevention and treatment strategies for cognitive decline.

## 1 Introduction

The devastating famine that swept across China during the 1960s represents a unique historical event that has profound implications for understanding the long-term consequences of early-life adversity on human health and development (1, 2). This period of widespread food scarcity provides a natural experiment to investigate the intricate relationship between nutritional deprivation during critical developmental windows and subsequent health outcomes. Among these outcomes, cognitive function, a vital aspect of human well-being and quality of life, has garnered significant attention due to its sensitivity to environmental during early life (3, 4).

Previous research has consistently demonstrated that adverse conditions during fetal, including malnutrition, can have lasting effects on physical growth, metabolic health, and even mental health (5–12). However, the specific impact of famine exposure during fetal life, a particularly vulnerable period of rapid brain development, on adult cognitive function remains controversial. Research by Li et al. in Heilongjiang Province, China, failed to find a significant association between parental prenatal famine exposure and offspring’s cognitive function in adulthood, casting doubt on the direct effect of prenatal famine (13). However, contrasting findings emerged from Chao et al. across different regions in China, which reported a correlation between fetal famine exposure and reduced Mini-Mental State Examination (MMSE) scores, emphasizing the potential for overall and specific cognitive declines associated with intrauterine famine exposure (14). Further complexity arises from the study conducted by Arage et al. (15) on the Ethiopian population, which has observed a statistical association between postnatal famine exposure and adult cognitive function, but not between prenatal famine exposure and adult cognitive function, suggesting that early childhood learning and experiences hold a relatively significant role in optimizing brain development postnatally. Meanwhile, Wiegersma’s research in the Netherlands underscored the long-lasting implications of prenatal famine exposure, linking it to increased self-reported cognitive problems at age 72 and gender-specific differences in healthcare utilization, highlighting the functional consequences of prenatal malnutrition that manifest with aging (16).

These conflicting and nuanced findings underscore the need for a comprehensive analysis that disentangles the unique and cumulative effects of famine exposure across different life stages on adult cognitive function. By leveraging the rich longitudinal data available in the China Health and Retirement Longitudinal Study (CHARLS) database, we aim to categorize individuals based on their famine exposure status during fetal life, preschool years, school-age years, and those unexposed, and to clarify the specific contribution of early-life famine exposure to cognitive outcomes in adulthood, with a particular focus on the timing of exposure.

## 2 Materials and methods

### 2.1 Study population and design

The CHARLS database is a national social survey aimed at collecting longitudinal data on the health, economic status, family structure, and retirement conditions of the Chinese population aged 45 and above. The project employs a multistage sampling design, using Probability Proportionate to Size Sampling (PPS) at both the county/district and village/residential levels to ensure that the selected sample is nationally representative (17). A baseline survey was conducted in 2011 (wave 1) among 17,708 participants recruited from 450 villages and residential communities in 28 provinces of China, followed by follow-up assessments every 2 to 3 years. During the CHARLS 2015 (wave 3) survey, participants were followed up for 4 years. The project has received approval from the Peking University Ethics Committee and obtained informed consent from all participants. The cohort study utilized participants’ data who underwent a baseline survey in 2011 and follow-up surveys in 2013 and 2015. Due to variations in the specific start and end dates of famines across different regions of China, and to minimize misclassification of the famine exposure period, the following inclusion criteria based on birth dates were established: (1) born between January 1, 1950, and December 31, 1957; (2) born between January 1, 1959, and December 31, 1962; and (3) born between January 1, 1964, and December 31, 1967. This classification standard is based on previous research (18).

### 2.2 Grouping by famine exposure period and severity

In this study, all birth dates are according to the Western calendar. Based on the relationship between birth dates and the years of the famine, subjects born between January 1, 1950, and December 31, 1953, were classified as the school-age exposed group; those born between January 1, 1954, and December 31, 1957, as the preschool exposed group; those born between January 1, 1959, and December 31, 1962, as the fetal exposed group; and those born between January 1, 1964, and December 31, 1967, as the non-exposed group.

Since the severity of the famine varied by region, based on previous research (19), excess mortality rates were used to define the severity of the famine. The excess mortality rate is calculated as the percentage change from the highest mortality rate in each province from 1959 to 1961 to the average mortality rate from 1956 to 1958. Provinces with an excess mortality rate exceeding 50% were defined as severely affected famine areas, while those with an excess mortality rate below 50% were defined as less severely affected famine areas. Mortality data for the provinces from 1956 to 1961 were sourced from the National Bureau of Statistics.

### 2.3 Assessment of cognitive function

Cognitive function was measured through two dimensions (20, 21). The first dimension is called episodic memory, which includes immediate word recall and delayed word recall. Immediate word recall involves the subjects immediately repeating as many words as possible from a list of 10 Chinese nouns read by the interviewer, without considering the order of the words (total score of 10). Delayed word recall requires the subjects to repeat as many words as possible from the same list after a delay of 4 to 10 min (total score of 10). Episodic memory scores were calculated as the average of the immediate word recall test and the delayed word recall test scores (ranging from 0 to 10) (22). The second dimension assesses the subjects’ executive function using questions from the Telephone Interview of Cognitive Status (TICS), which includes orientation ability, calculation ability and visuospatial ability. Orientation ability is assessed by asking the subjects to state the current date (including year, month, day, season, and the day of the week, for a total score of 5). Calculation ability is measured by having the subjects subtract 7 consecutively from 100 for five times (total score of 5). Visuospatial ability is assessed by showing the subjects a picture of two overlapping pentagons and asking them to draw a similar figure (total score of 1). Executive function scores were calculated as the sum of the three scores mentioned above (ranging from 0 to 11). The global cognitive score is the sum of the scores from these two dimensions (0–21 points), with higher scores indicating better cognitive function. Average cognitive score was calculated as the average of the cognitive function scores measured between 2011 and 2015.

### 2.4 Covariates

Based on the 2011 CHARLS questionnaire, trained interviewers collected demographic information, including age, gender, education level, marital status, and residence place. Education level was categorized into three groups: “illiterate,” “junior high school” and “high school or above.” Marital status was classified into “married/cohabitating” and “Others,” which including widowed, separated, divorced, and never married individuals. Residence place was divided into “rural” and “urban.” The assessment of social activities was based on the participants’ activities over the past month. Interviewers inquired “Have you done any of these activities in the last month?”. Participants were defined as “active” if they engaged in at least one activity; otherwise, they were defined as “inactive” (23). Drinking status was categorized into “drink but less than once a month,” “drink more than once a month” and “none of these” based on the frequency of drinking in the past year (24). Smoking status was classified as “current smoker,” “former smoker” and “never” (24). Hypertension was defined as a self-reported history or an average systolic blood pressure ≥ 140 mmHg or diastolic blood pressure ≥ 90 mmHg measured three times (25). Diabetes was defined as a self-reported history or fasting blood glucose ≥ 7.0 mmol/L or HbA1c ≥ 6.5% (26). Depression status was assessed using the Center for Epidemiologic Studies Depression Scale (CESD-10), with a CESD-10 score ≥ 12 indicating the presence of depression (27).

### 2.5 Statistical analysis

All analyses were conducted in R (version 4.4.1). Continuous data were expressed as mean ± standard error, and categorical variables were expressed as number (percentage). One-Way ANOVA was used for normally distributed continuous variables, the Kruskal-Wallis Rank Sum Test was used for non-normally distributed continuous variables, and the Chi-Squared Test was used for categorical variables to test for differences between different famine exposure periods. The relationship between famine exposure periods, famine severity, and cognitive function was analyzed using multiple linear regression models. Three models were established: the crude model, which did not adjust for any covariates; model 1, adjusted for age, gender, marital status, education level, residence place; and model 2, which further adjusted for social activity, drinking status, smoking status, depression, hypertension, DM, and famine exposure period based on model 1. Regression results were presented as regression coefficients (*β*) and 95% confidence intervals (CI). According to the Central Limit Theorem (28), the sample mean approximates a normal distribution when the sample size is sufficiently large. The Anderson-Darling Test was used to examine the normality of the regression residuals in the linear regression models. The results showed that the regression residuals of all models were approximately normally distributed, indicating that the analysis results are stable and reliable. Additionally, subgroup analyses were conducted based on gender, education level, marital status, residence place, social activity, drinking status, smoking status, depression, hypertension, and DM, and interactions were calculated to explore the variation in the effect of famine exposure periods on cognitive function across different subgroups. A two-sided *p* < 0.05 was considered statistically significant.

## 3 Results

### 3.1 Sample characteristics

**Figure 1:**
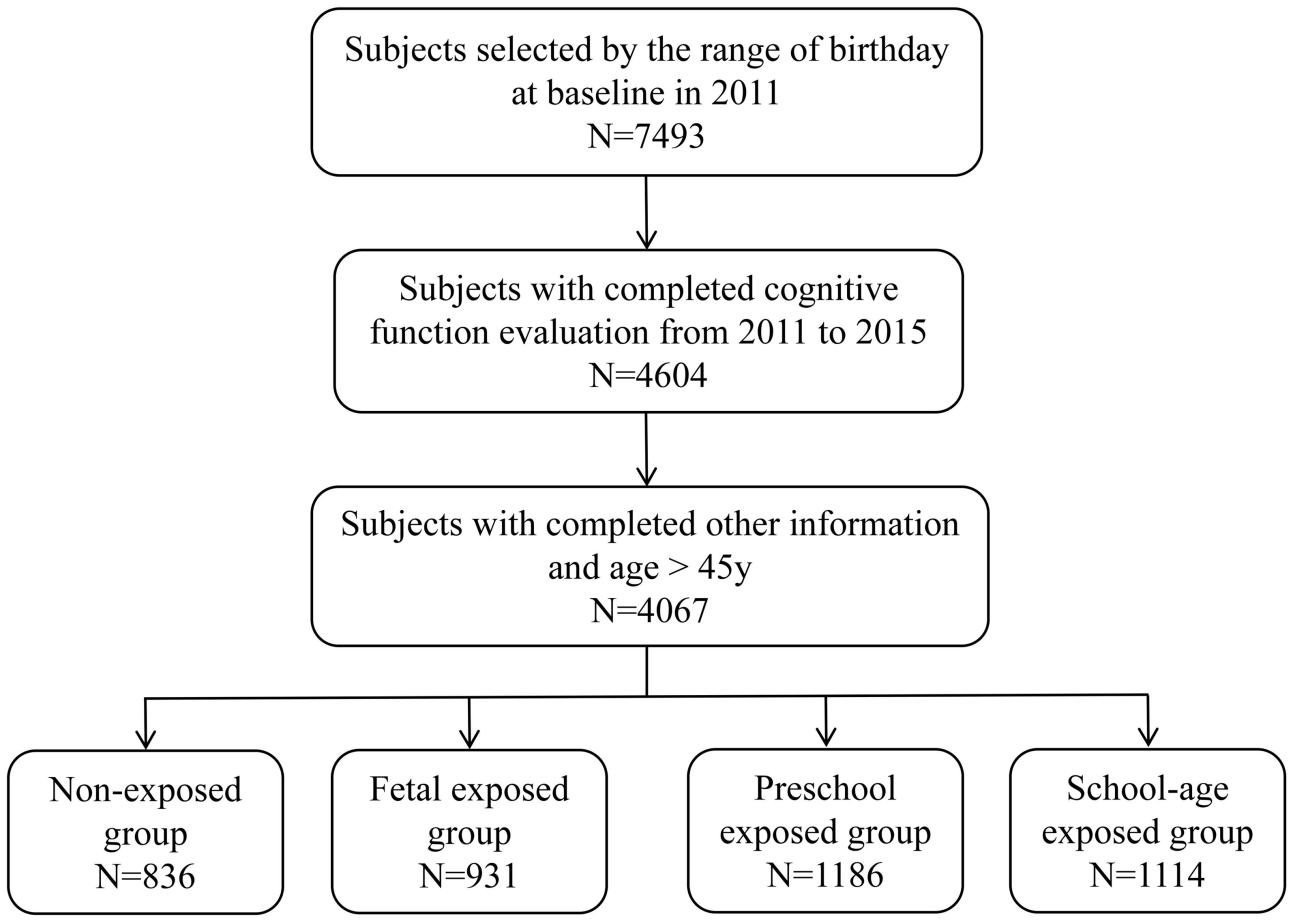
Flowchart on the sample selection of the famine cohort at each step.

Based on birth dates, a total of 7,493 participants were included in the study. After excluding participants with missing values in cognitive function and other information, 4,067 participants were included in the final analysis (Figure 1). Table 1 shows the baseline characteristics. The participants were divided into four groups based on famine exposure period: non-exposed group (*n* = 836), fetal exposed group (*n* = 931), preschool exposed group (*n* = 1,186) and school-age exposed group (*n* = 1,114). The mean age of the 4,067 participants was 53.51 ± 5.05 years, with 49.57% being female, and 38.78% living in severely affected famine areas. The four famine exposure subgroups were similar in terms of residence place, drinking status, and famine severity. Compared to the non-exposed group, the cognitive function scores in the fetal exposed group, preschool exposed group and school-age exposed group were all decreased to varying degrees (Table 2).

**Table 1:** Basic characteristics of study population according to exposure period to the Chinese famine.

Characteristics	Total (*n* = 4,067)	Non-exposed group (*n* = 836)	Fetal exposed group (*n* = 931)	Preschool exposed group (*n* = 1,186)	School-age exposed group (*n* = 1,114)	*P*-value^a^
Age, mean ± SD, year	53.51 ± 5.05	46.17 ± 0.77	50.33 ± 1.19	55.60 ± 1.13	59.46 ± 1.11	<0.0001
Gender, *n* (%)						<0.0001
Female	2016 (49.57)	482 (57.66)	484 (51.99)	556 (46.88)	494 (44.34)	
Male	2051 (50.43)	354 (42.34)	447 (48.01)	630 (53.12)	620 (55.66)	
Marital status, *n* (%)						<0.01
Married/cohabitating	3,836 (94.32)	805 (96.29)	879 (94.41)	1,119 (94.35)	1,033 (92.73)	
Others	231 (5.68)	31 (3.71)	52 (5.59)	67 (5.65)	81 (7.27)	
Social activity, *n* (%)						<0.0001
Inactive	1820 (44.75)	335 (40.07)	386 (41.46)	554 (46.71)	545 (48.92)	
Active	2,247 (55.25)	501 (59.93)	545 (58.54)	632 (53.29)	569 (51.08)	
Education, *n* (%)						<0.0001
Illiterate	490 (12.05)	42 (5.02)	74 (7.95)	173 (14.59)	201 (18.04)	
Junior high school	2,908 (71.50)	679 (81.22)	596 (64.02)	800 (67.45)	833 (74.78)	
High school or above	669 (16.45)	115 (13.76)	261 (28.03)	213 (17.96)	80 (7.18)	
Residence, *n* (%)						0.93
Rural	2,403 (59.09)	496 (59.33)	543 (58.32)	699 (58.94)	665 (59.69)	
Urban	1,664 (40.91)	340 (40.67)	388 (41.68)	487 (41.06)	449 (40.31)	
Drinking status, *n* (%)						0.29
Drink but less than once a month	382 (9.39)	83 (9.93)	93 (9.99)	107 (9.02)	99 (8.89)	
Drink more than once a month	1,163 (28.60)	210 (25.12)	267 (28.68)	352 (29.68)	334 (29.98)	
None of these	2,522 (62.01)	543 (64.95)	571 (61.33)	727 (61.30)	681 (61.13)	
Smoking status, *n* (%)						<0.0001
Current smoker	1,356 (33.34)	229 (27.39)	305 (32.76)	430 (36.26)	392 (35.19)	
Former smoker	328 (8.06)	45 (5.38)	50 (5.37)	104 (8.77)	129 (11.58)	
Never	2,383 (58.59)	562 (67.22)	576 (61.87)	652 (54.97)	593 (53.23)	
Hypertension, *n* (%)						<0.0001
No	2,777 (68.28)	662 (79.19)	654 (70.25)	809 (68.21)	652 (58.53)	
Yes	1,290 (31.72)	174 (20.81)	277 (29.75)	377 (31.79)	462 (41.47)	
DM, *n* (%)						<0.0001
No	3,598 (88.47)	763 (91.27)	829 (89.04)	1,048 (88.36)	958 (86.00)	
Yes	469 (11.53)	73 (8.73)	102 (10.96)	138 (11.64)	156 (14.00)	
Depression, *n* (%)						<0.01
No	2,802 (68.90)	598 (71.53)	646 (69.39)	839 (70.74)	719 (64.54)	
Yes	1,265 (31.10)	238 (28.47)	285 (30.61)	347 (29.26)	395 (35.46)	
Famine severity, *n* (%)						0.19
Less severely affected famine area	2,490 (61.22)	497 (59.45)	597 (64.12)	722 (60.88)	674 (60.50)	
Severely affected famine area	1,577 (38.78)	339 (40.55)	334 (35.88)	464 (39.12)	440 (39.50)	

SD, standard deviation; DM, diabetes mellitus.

Values are means ± SDs for continuous variables and *n* (%) for categorical variables.

^a^Analysis of variance and the Chi-squared test were used to test for difference across groups.

**Table 2:** Cognitive descriptions of study population according to exposure period to the Chinese famine.

Cognitive descriptions	Total (*n* = 4,067)	Non-exposed group (*n* = 836)	Fetal exposed group (*n* = 931)	Preschool exposed group (*n* = 1,186)	School-age exposed group (*n* = 1,114)	*P*-value^a^
Cognitive score at baseline in 2011, mean ± SD						
Global cognition	12.45 ± 3.23	13.12 ± 3.06	12.67 ± 3.15	12.30 ± 3.18	11.90 ± 3.37	<0.0001
Episode memory	4.01 ± 1.59	4.42 ± 1.63	4.10 ± 1.57	3.90 ± 1.56	3.75 ± 1.55	<0.0001
Executive function	8.44 ± 2.41	8.71 ± 2.24	8.57 ± 2.33	8.41 ± 2.38	8.16 ± 2.58	<0.0001
Cognitive score at visit two in 2013, mean ± SD						
Global cognition	12.38 ± 3.30	13.08 ± 3.07	12.54 ± 3.31	12.22 ± 3.31	11.89 ± 3.34	<0.0001
Episode memory	4.14 ± 1.59	4.54 ± 1.57	4.27 ± 1.64	4.05 ± 1.57	3.81 ± 1.51	<0.0001
Executive function	8.24 ± 2.46	8.54 ± 2.33	8.27 ± 2.42	8.17 ± 2.47	8.08 ± 2.55	<0.001
Cognitive score at visit three in 2015, mean ± SD						
Global cognition	12.06 ± 3.36	12.87 ± 3.11	12.26 ± 3.27	11.90 ± 3.37	11.45 ± 3.48	<0.0001
Episode memory	3.94 ± 1.63	4.42 ± 1.64	4.09 ± 1.61	3.85 ± 1.57	3.56 ± 1.60	<0.0001
Executive function	8.12 ± 2.48	8.45 ± 2.31	8.17 ± 2.40	8.05 ± 2.52	7.89 ± 2.61	<0.0001
Average cognitive score during 2011–2015, mean ± SD						
Global cognition	12.30 ± 2.72	13.03 ± 2.50	12.49 ± 2.66	12.14 ± 2.72	11.75 ± 2.78	<0.0001
Episode memory	4.03 ± 1.24	4.46 ± 1.24	4.15 ± 1.25	3.93 ± 1.19	3.70 ± 1.18	<0.0001
Executive function	8.27 ± 1.94	8.57 ± 1.79	8.33 ± 1.85	8.21 ± 1.97	8.04 ± 2.06	<0.0001

Abbreviations: SD, standard deviation.

Values are means ± SDs for continuous variables.

^a^Analysis of variance and the Chi-squared test were used to test for difference across groups.

### 3.2 Association of famine exposure in early life stage with cognitive function in adulthood

Table 3 illustrates the relationship between different famine exposure periods and cognitive function at baseline in 2011. In the univariate analysis, nearly all exposed groups showed a decline in cognitive function compared to the non-exposed group, with statistically significant differences. However, in Model 2, which adjusted for all confounding factors, only the fetal exposed group still exhibited significant differences in global cognition, episode memory and executive function compared to the non-exposed group (global cognition, *β* = −0.63, [95% CI: −1.07, −0.18]; episode memory, *β* = −0.28, [95% CI: −0.51, −0.05]; executive function, *β* = −0.35, [95% CI: −0.68, −0.02]). No significant differences were observed in cognitive function scores for other famine exposed periods.

**Table 3:** Multivariate regression analysis of the association between famine exposed period and cognitive function at baseline in 2011.

Cognitive score at baseline in 2011	Crude model		Model 1		Model 2	
	β (95% CI)	*P*-value	β (95% CI)	*P*-value	β (95% CI)	*P*-value
Global cognition						
Non-exposed group	Ref		Ref		Ref	
Fetal exposed group	−0.45 (−0.75, −0.15)	0.003	−0.65 (−1.10, −0.20)	0.005	−0.63 (−1.07, −0.18)	0.01
Preschool exposed group	−0.82 (−1.10, −0.53)	<0.0001	−0.75 (−1.59, 0.09)	0.08	−0.76 (−1.58, 0.07)	0.07
School-age exposed group	−1.22 (−1.51, −0.93)	<0.0001	−0.92 (−2.09, 0.24)	0.12	−0.90 (−2.03, 0.24)	0.12
Episode memory						
Non-exposed group	Ref		Ref		Ref	
Fetal exposed group	−0.31 (−0.46, −0.16)	<0.0001	−0.29 (−0.53, −0.06)	0.01	−0.28 (−0.51, −0.05)	0.02
Preschool exposed group	−0.52 (−0.66, −0.38)	<0.0001	−0.27 (−0.71, 0.17)	0.23	−0.27 (−0.70, 0.16)	0.22
School-age exposed group	−0.67 (−0.81, −0.53)	<0.0001	−0.23 (−0.83, 0.37)	0.45	−0.21 (−0.81, 0.38)	0.48
Executive function						
Non-exposed group	Ref		Ref		Ref	
Fetal exposed group	−0.14 (−0.36, 0.08)	0.22	**−0.36 (−0.70, −0.02)**	0.04	−0.35 (−0.68, −0.02)	0.04
Preschool exposed group	−0.30 (−0.51, −0.08)	0.01	−0.48 (−1.11, 0.15)	0.14	−0.49 (−1.11, 0.14)	0.12
School-age exposed group	−0.55 (−0.76, −0.33)	<0.0001	−0.69 (−1.56, 0.17)	0.12	−0.68 (−1.54, 0.17)	0.12

Crude model, unadjusted.

Model 1, adjusted for age, gender, marital status, education level, residence place.

Model 2, additionally adjusted for social activity, drinking status, smoking status, depression, hypertension, DM and famine severity.

CI, confidence intervals; DM, diabetes mellitus.

Table 4 shows the association between different famine exposure periods and average cognitive scores during 2011–2015. The results indicate that in the fully adjusted model, the fetal-exposed group showed significant declines in global cognition (*β* = −0.60, [95% CI: −0.95, −0.25]), episodic memory (*β* = −0.25, [95% CI: −0.42, −0.07]), and executive function (*β* = −0.36, [95% CI: −0.61, −0.10]) compared to the non-exposed group. However, after adjusting for potential confounders, the changes of preschool and school-age exposures were no longer significant.

**Table 4:** Multivariate regression analysis of the association between famine exposed period and average cognitive score during 2011–2015.

Average cognitive score during 2011–2015	Crude model		Model 1		Model 2	
	β (95% CI)	*P*-value	β (95% CI)	*P*-value	β (95% CI)	*P*-value
Global cognition						
Non-exposed group	0.00	Ref	0.00	Ref	0.00	Ref
Fetal exposed group	−0.54 (−0.79, −0.29)	<0.0001	−0.63 (−0.99, −0.27)	<0.001	−0.60 (−0.95, −0.25)	<0.001
Preschool exposed group	−0.88 (−1.12, −0.64)	<0.0001	−0.52 (−1.19, 0.15)	0.13	−0.52 (−1.18, 0.13)	0.12
School-age exposed group	−1.28 (−1.52, −1.04)	<0.0001	−0.56 (−1.48, 0.37)	0.24	−0.54 (−1.43, 0.36)	0.24
Episode memory						
Non-exposed group	0.00	Ref	0.00	Ref	0.00	Ref
Fetal exposed group	−0.30 (−0.42, −0.19)	<0.0001	−0.25 (−0.43, −0.08)	0.005	−0.25 (−0.42, −0.07)	0.01
Preschool exposed group	−0.53 (−0.63, −0.42)	<0.0001	−0.19 (−0.51, 0.14)	0.26	−0.19 (−0.51, 0.13)	0.24
School-age exposed group	−0.76 (−0.87, −0.65)	<0.0001	−0.19 (−0.64, 0.26)	0.41	−0.18 (−0.63, 0.26)	0.42
Executive function						
Non-exposed group	0.00	Ref	0.00	Ref	0.00	Ref
Fetal exposed group	−0.23 (−0.41, −0.05)	0.01	−0.37 (−0.63, −0.12)	0.005	−0.36 (−0.61, −0.10)	0.01
Preschool exposed group	−0.36 (−0.53, −0.18)	<0.001	−0.34 (−0.82, 0.15)	0.17	−0.33 (−0.80, 0.14)	0.17
School-age exposed group	−0.52 (−0.70, −0.35)	<0.001	−0.37 (−1.03, 0.29)	0.28	−0.35 (−1.00, 0.30)	0.29

Crude model, unadjusted.

Model 1, adjusted for age, gender, marital status, education level, residence place.

Model 2, additionally adjusted for social activity, drinking status, smoking status, depression, hypertension, DM and famine severity.

CI, confidence intervals; DM, diabetes mellitus.

**Figure 2:**
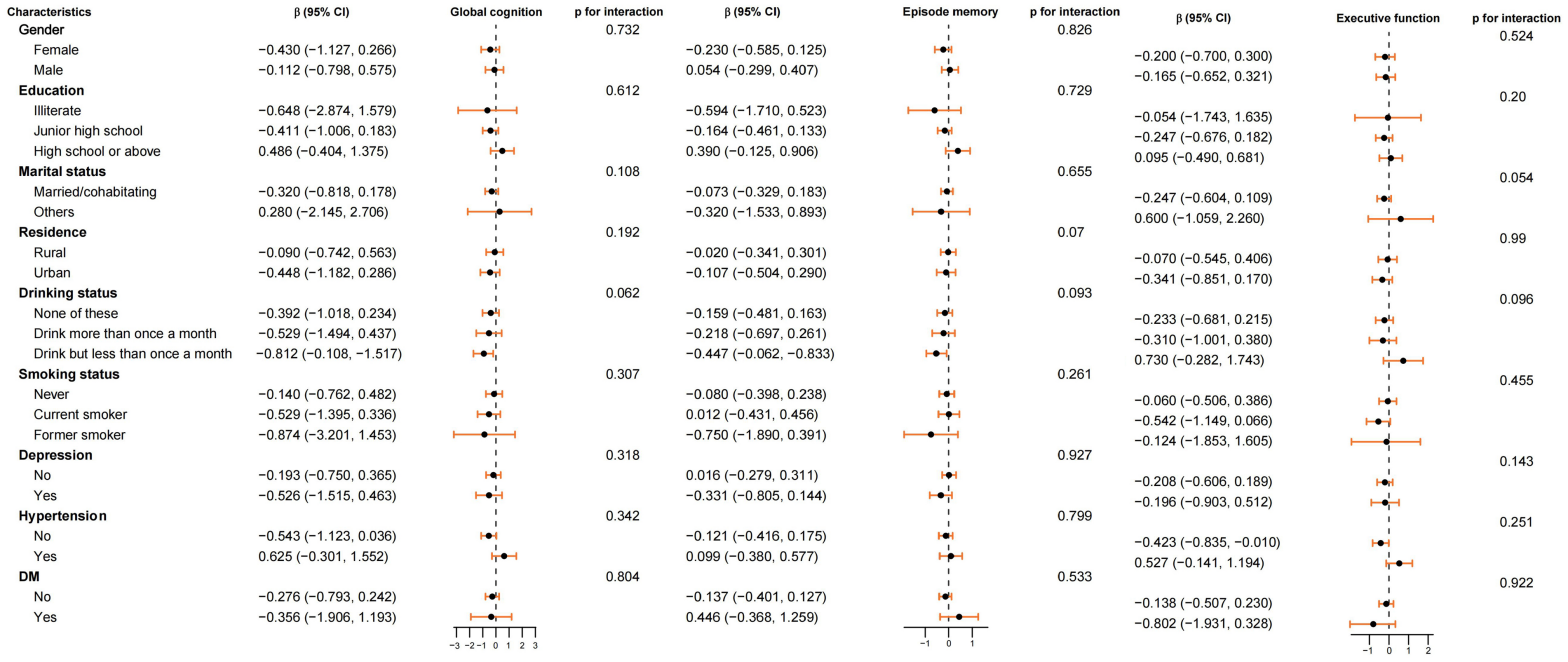
Subgroup analyses of the association between famine exposed period and average cognitive score during 2011–2015 (global cognition, episode memory and executive function) in non-exposed group and fetal exposed group. Model adjusted for age, gender, marital status, education level, residence place, social activity, drinking status, smoking status, depression, hypertension, DM and famine severity. Stratification variables were not adjusted in corresponding models.

Compared to non-exposed group, the association between the fetal exposure group and average cognitive scores during 2011–2015 (including global cognition, episodic memory, and executive function) were analyzed in subgroups based on gender, education level, marital status, residence, drinking status, smoking status, depression, hypertension and DM (Figure 2). The results indicated that the impact of fetal famine exposure on cognitive function was consistent across all subgroups, with no significant interactions observed (*P* for interaction > 0.05).

Table 5 summarizes the association between famine exposure, stratified by famine severity, and cognitive function. After adjusting for all covariates, compared to the non-exposed group, the fetal exposed group in severely affected famine areas showed significantly lower global cognition (*β* = −0.64, [95% CI: −1.09, −0.19]), episodic memory (*β* = −0.35, [95% CI: −0.64, −0.06]), and executive function (*β* = −0.47, [95% CI: −0.80, −0.14]). No significant associations were observed between the preschool exposed group or the school-age exposed group and cognitive function in severely affected famine areas. In less severely affected famine areas, compared to the non-exposed group, the fetal exposed group had significantly lower executive function (*β* = −0.41, [95% CI: −0.62, −0.19]), with a borderline effect on global cognition (*β* = −0.57, [95% CI: −1.13, 0.00]), and no significant effect on episodic memory. No significant interactions were observed between the famine exposed groups (fetal, preschool, or school-aged vs. non-exposed) and areas (severely affected vs. less severely affected) in the average cognitive scores during 2011–2015.

**Table 5:** Association of famine exposure and average cognitive score during 2011–2015 by famine severity.

Average cognitive score during 2011–2015	Crude model		Model 1		Model 2	
	β (95% CI)	*P*-value	β (95% CI)	*P*-value	β (95% CI)	*P*-value
Global cognition						
Less severely affected famine area						
Non-exposed group	0.00	Ref	0.00	Ref	0.00	Ref
Fetal exposed group	−0.52 (−0.93, −0.12)	0.01	−0.59 (−1.16, −0.02)	0.04	−0.57 (−1.13, 0.00)	0.05
Preschool exposed group	−1.08 (−1.45, −0.70)	<0.0001	−0.76 (−1.85, 0.33)	0.17	−0.72 (−1.79, 0.35)	0.19
School-age exposed group	−1.55 (−1.93, −1.17)	<0.0001	−0.80 (−2.29, 0.69)	0.29	−0.64 (−2.10, 0.82)	0.39
Severely affected famine area						
Non-exposed group	0.00	Ref	0.00	Ref	0.00	Ref
Fetal exposed group	−0.56 (−0.88, −0.24)	<0.001	−0.71 (−1.17, −0.25)	0.002	−0.64 (−1.09, −0.19)	0.01
Preschool exposed group	−0.76 (−1.07, −0.45)	<0.0001	−0.48 (−1.33, 0.37)	0.27	−0.42 (−1.25, 0.41)	0.32
School-age exposed group	−1.10 (−1.41, −0.79)	<0.0001	−0.56 (−1.73, 0.62)	0.35	−0.50 (−1.65, 0.64)	0.39
*P* for interaction between area and group: 0.072^a^						
Episode memory						
Less severely affected famine area						
Non-exposed group	0.00	Ref	0.00	Ref	0.00	Ref
Fetal exposed group	−0.28 (−0.43, −0.14)	<0.0001	−0.20 (−0.43, 0.02)	0.07	−0.17 (−0.39, 0.05)	0.12
Preschool exposed group	−0.47 (−0.61, −0.33)	<0.0001	−0.06 (−0.46, 0.35)	0.79	−0.03 (−0.43, 0.37)	0.88
School-age exposed group	−0.64 (−0.78, −0.51)	<0.0001	0.02 (−0.55, 0.58)	0.96	0.04 (−0.51, 0.59)	0.89
Severely affected famine area						
Non-exposed group	0.00	Ref	0.00	Ref	0.00	Ref
Fetal exposed group	−0.34 (−0.53, −0.16)	<0.001	−0.36 (−0.64, −0.07)	0.02	−0.35 (−0.64, −0.06)	0.02
Preschool exposed group	−0.61 (−0.79, −0.44)	<0.0001	−0.43 (−0.97, 0.12)	0.13	−0.42 (−0.96, 0.12)	0.13
School-age exposed group	−0.93 (−1.10, −0.75)	<0.0001	−0.56 (−1.31, 0.19)	0.14	−0.52 (−1.26, 0.22)	0.17
*P* for interaction between area and group: 0.093^a^						
Executive function						
Less severely affected famine area						
Non-exposed group	0.00	Ref	0.00	Ref	0.00	Ref
Fetal exposed group	−0.18 (−0.47, −0.11)	<0.001	−0.41 (−0.64, −0.18)	0.03	−0.41 (−0.62, −0.19)	0.03
Preschool exposed group	−0.46 (−0.73, −0.19)	<0.001	−0.34 (−1.12, 0.44)	0.40	−0.30 (−1.07, 0.47)	0.45
School-age exposed group	−0.62 (−0.90, −0.35)	<0.0001	−0.24 (−1.31, 0.82)	0.66	−0.13 (−1.18, 0.93)	0.81
Severely affected famine area						
Non-exposed group	0.00	Ref	0.00	Ref	0.00	Ref
Fetal exposed group	−0.27 (−0.50, −0.04)	0.02	−0.51 (−0.84, −0.18)	0.003	−0.47 (−0.80, −0.14)	0.005
Preschool exposed group	−0.29 (−0.51, −0.07)	0.01	−0.43 (−1.04, 0.19)	0.17	−0.39 (−1.00, 0.21)	0.20
School-age exposed group	−0.46 (−0.68, −0.24)	<0.0001	−0.57 (−1.41, 0.27)	0.18	−0.54 (−1.38, 0.29)	0.20
*P* for interaction between area and group: 0.112^a^						

Crude model, unadjusted.

Model 1, adjusted for age, gender, marital status, education level, residence place.

Model 2, additionally adjusted for social activity, drinking status, smoking status, depression, hypertension and DM.

^a^Adjusted for the covariates in Model 2.

CI, confidence intervals; DM, diabetes mellitus.

## 4 Discussion

This study aimed to explore the relationship between different periods of famine exposure, the severity of famine, and cognitive function. Our findings revealed that individuals who experienced famine exposure during the fetal period exhibited significant declines in global cognition, episode memory and executive function upon reaching adulthood compared to those who were not exposed to famine, even after adjusting for multiple confounding factors. In contrast, preschool and school-age exposure did not demonstrate similar associations. Furthermore, stratified analyses by famine severity revealed that the adverse effects of fetal famine exposure on cognitive function were more pronounced in severely affected famine regions compared to less severely affected areas. This study highlights the significant impact of early nutritional adversity on brain development and long-term cognitive function, underscoring the crucial importance of adequate nutritional support during critical periods of growth.

In the context of the present study, several underlying mechanisms play a crucial role in explaining how prenatal nutrition critically shapes brain development and, consequently, cognitive function in adulthood. These mechanisms encompass epigenetic modifications, nutrient-mediated neuronal proliferation and differentiation, as well as the intricate interplay between nutrition and environmental exposure during sensitive periods of brain growth.

### 4.1 Epigenetic programming of brain development

A pivotal mechanism supporting the observed effects of early nutrition on cognitive outcomes is epigenetic regulation. Recent research underscores the role of DNA and histone methylation in mediating the influence of nutritional factors, such as choline and α-linolenic acid, on brain development (29–32). These epigenetic marks act as molecular switches, modulating gene expression patterns that are crucial for neural circuit formation, synaptic plasticity, and ultimately, cognitive abilities (29, 31). Notably, the sensitivity of the epigenome to early life exposures, including nutrition, suggests that even subtle changes during this period can have long-lasting consequences for the individual’s cognitive trajectory (33, 34). Furthermore, the transgenerational transmission of such epigenetic marks via the germline highlights the potential for intergenerational effects of early life nutrition on cognitive health (33, 34). By modulating epigenetic marks, early nutrition can potentially influence not only the immediate generation but also future generations, emphasizing the need for careful consideration of dietary choices during these pivotal life stages.

### 4.2 Nutrient-mediated neuronal proliferation and differentiation

Nutrients serve as essential components of neuronal growth and differentiation, playing a vital role in the rapid brain expansion observed during fetal (35–37). Deficiencies in key nutrients, including those involved in neurotransmitter synthesis, cell proliferation, DNA synthesis, and enzyme function, can impede these processes, leading to structural and functional alterations in the developing brain (38). For instance, inadequate choline supply during pregnancy has been linked to reduced neural progenitor proliferation and altered neuronal migration, both of which contribute to the formation of complex neural circuits (29). Similarly, insufficient ω-3 polyunsaturated fatty acids, like *α*-linolenic acid, impair synaptic development and plasticity, critical for learning and memory (31).

### 4.3 The intersection of nutrition and environmental exposures

The developing brain is particularly susceptible to the combined effects of nutrition and other environmental factors, such as stress or limited sensory stimulation (39, 40). Malnutrition, when coupled with adverse life experiences, can exacerbate the negative impact on cognitive development by disrupting multiple developmental pathways simultaneously. For instance, postnatal malnutrition not only affects the physical capacity to interact with the environment but also alters the emotional and motivational drives that govern exploratory behavior, further compromising language, motor, and social skill acquisition—all of which are fundamental to adult cognitive functioning (39, 40). The clinical research conducted by Wang et al. across various regions in China, and by Arage et al. (15) specifically targeting the Ethiopian population, provide additional support to this argument (14). This complex interplay underscores the importance of combined effects to optimizing early life conditions for optimal cognitive outcomes.

In addition to the biological mechanisms discussed, the role of education and sociocultural factors in modulating cognitive reserve requires particular attention in this famine-exposed population. Cognitive reserve theory posits that higher educational attainment and enriched cultural engagement may enhance neural plasticity and provide compensatory pathways to mitigate the effects of brain insults (41, 42). In our study, education level was adjusted as a covariate across all models, yet the persistent association between fetal famine exposure and cognitive deficits suggests that early nutritional deprivation may override the protective effects of education in this population. This observation aligns with neuroimaging studies demonstrating that early malnutrition induces structural brain alterations (e.g., reduced hippocampal volume and prefrontal cortical thickness) that are not fully compensated by later educational attainment (43, 44).

It is noteworthy that after adjusting for relevant confounding factors, our current study did not detect a direct statistical association between famine exposure during the preschool and school-age periods and cognitive function scores in adulthood. However, this could not ignore the potential long-term implications of such exposure in these population. On one hand, the absence of a significant finding may reflect the substantial improvements in economic, educational, and nutritional environments experienced by the study population, which may have mitigated the detrimental effects of early adversity on cognitive development to some extent (45–47). On the other hand, the assessment of cognitive function is a longitudinal and multidimensional process (48–52). While our study has adopted a more longitudinal approach by incorporating baseline cognitive scores from 2011 and averaging scores across 2011–2015, accompanied by multiple follow-ups to better capture the trajectory of cognitive development, it cannot discount the possibility that famine exposure during preschool and school-age years may significantly impact cognitive function in the more distant future. Furthermore, the current cognitive assessment methods employed may not fully capture the nuanced effects of famine exposure on specific cognitive domains or finer cognitive functions during these critical developmental periods. Consequently, future research should consider designing longer-term follow-ups and incorporating a more diverse range of cognitive assessment tools to gain a more comprehensive understanding of the complexity and dynamics of the long-term effects of nutritional and environmental exposures on cognitive development.

Additionally, our observations revealed that participants from severely famine-affected regions demonstrated marked deficits across multiple domains of cognitive function in comparison to those from less severely famine-stricken areas. Notably, even after adjusting for various potential confounding factors, the negative impact of severe famine on global cognition, episode memory and executive function remained significant, underscoring the pivotal role of famine exposure as an independent risk factor. This research finding carries profound implications for public health policy formulation and intervention design. Firstly, it is necessary to prioritize and strengthen investments in the nutritional security of children residing in regions affected by severe famine (53). Secondly, in light of the widespread effects of famine exposure on cognitive function, future research and interventions should focus on exploring effective strategies to mitigate or reverse these adverse consequences. These may encompass comprehensive measures such as nutritional supplementation, cognitive training, and mental health support (54–56).

Lastly, the further subgroup analysis deepened our comprehension of the intricacies surrounding the relationship between famine exposure and cognitive function. Intriguingly, regardless of participants’ gender, education level, marital status, residence, alcohol and smoking habits, depressive symptoms, hypertension or DM status, the detrimental effects of famine exposure on cognitive function remained consistent, without significant interactions observed. This discovery highlights the universal consistency of famine’s impact on cognitive function across diverse populations, unaffected by other prevalent health factors or lifestyle habits. Consequently, it may not be necessary to tailor policies and interventions solely to specific population groups or characteristics, but rather to adopt more universal strategies that encompass a broad spectrum of individuals (53).

Our current study has several limitations worth emphasizing. First, there is the issue of survival bias. Potential participants who were unable to participate due to health issues or who have already passed away as a result of famine exposure may not be included in our study, potentially limiting the accuracy of our findings. Second, despite our efforts to adjust for multiple covariates in the statistical analysis, there may still be unaccounted potential factors that could influence the relationship between famine exposure and cognitive function. These include factors such as genetics, early life environments, childhood nutrition, and parent–child bond, which may intricately interact with famine exposure and could have impacts on cognitive outcomes. Third, underexplored intergenerational effects represent another limitation. Our study has primarily focused on the direct effects of famine exposure on individuals, but it is now recognized that famine can also have transgenerational impacts. These effects, which may manifest in future generations through epigenetic mechanisms or other pathways, were not fully explored in our current study and represent an important direction for future research.

In summary, this study has uncovered significant negative impacts of famine exposure during early life stages on cognitive function in adulthood. This finding underscores the critical importance of early nutritional interventions, particularly in famine-prone regions. To improve the cognitive health of populations affected by famine, we recommend strengthening nutritional monitoring and intervention measures to ensure that children receive adequate nutritional support during their crucial developmental phases. Furthermore, governments and all sectors of society should increase investments in famine prevention and response efforts to mitigate the long-term health impacts of famine on populations.

## 5 Conclusion

Fetal famine exposure is negatively related to global cognition, episode memory and executive function. Severity of famine during fetal is associated with reduced cognitive performance in adulthood.

## Data Availability

Publicly available datasets were analyzed in this study. This data can be found at: http://charls.pku.edu.cn/pages/data/111/zh-cn.html.
